# It takes a pirate to know one: ethical hackers for healthcare cybersecurity

**DOI:** 10.1186/s12910-022-00872-y

**Published:** 2022-12-09

**Authors:** Giorgia Lorenzini, David Martin Shaw, Bernice Simone Elger

**Affiliations:** 1 Institute for Biomedical Ethics, Faculty of Medicine, University of Basel, Bernoullistrasse 28, 4056 Basel, Switzerland; 2 Care and Public Health Research Institute, Faculty of Health, Medicine and Life Sciences, Maastricht University, Maastricht, The Netherlands; 3 Center of Legal Medicine, Faculty of Medicine, University of Geneva, Geneva, Switzerland

**Keywords:** Cybersecurity, Hacker ethics, Health data, Penetration test

## Abstract

Healthcare cybersecurity is increasingly targeted by malicious hackers. This sector has many vulnerabilities and health data is very sensitive and valuable. Consequently, any damage caused by malicious intrusions is particularly alarming. The consequences of these attacks can be enormous and endanger patient care. Amongst the already-implemented cybersecurity measures and the ones that need to be further improved, this paper aims to demonstrate how penetration tests can greatly benefit healthcare cybersecurity. It is already proven that this approach has enforced cybersecurity in other sectors. However, it is not popular in healthcare since many prejudices still surround the hacking practice and there is a lack of education on hackers’ categories and their ethics. The present analysis aims to comprehend what hacker ethics is and who ethical hackers are. Currently, hacker ethics has the status of personal ethics; however, to employ penetration testers in healthcare, it is recommended to draft an official code of ethics, comprising principles, standards, expectations, and best practices. Additionally, it is important to distinguish between malicious hackers and ethical hackers. Amongst the latter, penetration testers are only a sub-category. Acknowledging the subtle differences between ethical hackers and penetration testers allows to better understand why and how the latter can offer their services to healthcare facilities.

## Background

Cybersecurity is a major concern in almost every context nowadays, and our reliance on interconnected technologies leaves companies and institutions extremely vulnerable to hackers’ attacks. Recent attacks have made it clear that every system has some vulnerabilities, and it is simply a matter of time until some malicious hacker exploits them. Particularly in the healthcare context, cyber threats are becoming increasingly common and a growing concern [1–3]. Healthcare has come, and is greatly encouraged, to rely on digital technologies, such as electronic health records (EHR), wearable devices, and artificial intelligence (AI) tools, which further augment vulnerabilities [2, 4]. Since the outbreak of the COVID-19 pandemic, cyberattacks on healthcare facilities have intensified and they have put additional strain on the already-overwhelmed healthcare industry [4]. Amongst other examples, in March 2020 the Czech Brno University Hospital, a COVID-19 testing facility, was targeted by hackers, forcing its entire IT network to shut down and causing the cancellation of all surgeries; during the same month, the World Health Organization saw the creation of a spoof site that mimicked their own, aiming to steal employees’ passwords [4–6]. There are numerous other examples, however, many cyberattacks are undetected or unreported and only a minority of them are publicly disclosed [7]. Accordingly, it is difficult to precisely assess the prevalence of these attacks and their consequences, as well as to intervene promptly. This underreporting conveys a false sense of security while failing to raise the necessary awareness to take protective measures. However, some jurisdictions have imposed obligations for notifying cyber incidents and data breaches; two examples are the Cyber Incident Reporting for Critical Infrastructure Act in the US and Article 33 of the European General Data Protection Regulation (GDPR) [8, 9]. Further awareness and cyber-hygiene measures are nonetheless needed [4].

Cyber threats are particularly severe in healthcare for two reasons: the vulnerabilities of this sector and the dramatic consequences that can result from their penetration. Indeed, cyberattacks can negatively affect public trust, damage critical equipment, and threaten human lives [10]. When a cyberattack occurs, there is little room for negotiation without putting patients’ care at risk [11]. Moreover, it can be profitable to target the healthcare industry: financial gain can be significant as health data is very valuable [3, 7].

This paper aims to show how penetration tests (pen-tests) conducted by ethical hackers can be beneficial for healthcare cybersecurity. It is already established that this approach has enforced cybersecurity in other sectors, where vulnerabilities have been located and the defence system has been reinforced. Pen-testers “have a valuable role to play in probing hardware, software or websites to look for weaknesses” [12]. In addition to contributing a lot through pen-tests, they can help address the labour shortage affecting the sector [13]. However, it seems that this service is not commonly provided to healthcare facilities. This could partly depend on the fears and prejudices towards the hacking practice, on the lack of a clear, and official, code of ethics for this profession, and on the limited financial resources that these facilities can devote to cybersecurity. The present analysis addresses the first difficulty by illustrating how pen-tests are a serious service offered by respectable and reliable companies and professionals. The second difficulty is addressed by presenting hacker ethics: the description of this ethics may contribute to rise the awareness necessary to effectively collaborate with ethical hackers, and pen-testers in particular.

## Special status of healthcare cybersecurity

A combination of factors renders healthcare particularly exposed to cyberattacks while being profitable for malicious hackers. In this section, both the vulnerabilities and the value of health data will be explored.

### Health data

Health data are considered one of the most personal types of information by citizens, so unauthorized access is particularly alarming [14]. Health data breaches can have serious consequences for individuals’ privacy as they can cause stigma, discrimination, and embarrassment to patients. Furthermore, they can impact patients’ jobs, insurance and economic status, and family relations [1]. This is especially true in the case of well-known individuals, such as politicians and celebrities, or in the case of already vulnerable and stigmatized populations. Nonetheless, the negative consequences of health data breaches are potentially serious for every individual.

Since health data document intimate personal information that cannot be reset, unlike other types of personal sensitive information (e.g. credit cards), they are interesting and profitable for the black market [15]. Often, these records contain enough information to open bank accounts, obtain loans, or acquire an identity document [7]. Therefore, health records can be worth more than credit card information and they allow identity thieves to create convincing identities [16]. When the records do not contain sufficient personal information, they can anyhow be valuable for the black market as they allow access to prescription drugs [10].

Health data are obviously vital also for healthcare: when hackers launch ransomware attacks and lock access to this data, the entire workflow is halted. Ransomware is a malicious program that encrypts the information stored on the servers, rendering them inaccessible to the staff. Usually, the encryption is followed by a ransom demand for the decryption keys [4]. It is not possible to check a patient’s blood type, surgeries are cancelled, and everything has to be noted by hand [17]. One exemplary case is the University of Vermont (UVM) Medical Center, which was hit by a ransomware attack during the COVID-19 pandemic in October 2020, when an employee opened a phishing email [18]. The attack caused the shutdown of all internet connections, precluding access to patients’ EHR. Unable to communicate, they sent employees to buy walkie-talkies. For nearly a month, the UVM Medical Center could not use EHR and other digital tools. For days their staff could not access patients’ appointments. Many surgeries were rescheduled and cancer patients were re-addressed to other facilities for radiation treatment [19]. When the system is finally accessible again, all the handwritten data noted during the time the system was inaccessible has to be reported back to the computer. This is a time-consuming activity and it can take months before returning to the pre-attack situation. Therefore, protecting health data is essential for ensuring patients’ safety.

### Vulnerabilities

The healthcare industry has many vulnerabilities that can be exploited by malicious hackers. Longstanding insufficient investment in IT is one first factor [10]. For example, legacy software, such as Windows XP, is still frequently used although this operating system is no longer supported by security updates. This makes it easier for malicious hackers to exploit vulnerabilities [10]. The situation is further exacerbated by the shortage of cybersecurity experts; in order to attract them, companies offer them exceedingly competitive salaries that healthcare organisations often cannot match [7, 13, 20]. Prioritising patient care, the healthcare sector lacks the resources necessary to establish a solid cyber defence. Therefore, in several healthcare facilities cybersecurity has been neglected to various degrees.

A second factor accounting for healthcare vulnerability is the implementation of interconnected technologies that, while enabling remote and distributed access to care, constitute an opportunity for intrusion [21]. The cybersecurity issue is one of the challenges posed by the introduction of new technologies in healthcare.^1^ For example, a novel concern is the malicious intrusions into medical devices such as pacemakers and insulin pumps: as an ethical hacker demonstrated, it is theoretically possible to remotely hack a Medtronic insulin pump to deliver a lethal dose of insulin, with potentially disastrous consequences for the patient [22]. While there are no reports of attacks on insulin pumps, in 2015 there has been a massive cyberattack targeting medical devices, known as MEDJACK [23]. Unbeknownst to hospitals’ staff, diagnostic equipment (MRI machines and CT scanners), therapeutic equipment (infusion pumps), and life support equipment (ventilators) were compromised [24].

## Penetration tests

Cybersecurity experts design their techniques based on assumptions about malicious hackers’ behaviour. However, without actual knowledge of the hacking practice and motivations, these assumptions often reveal unrealistic expectations and might overlook important factors [25]. This is where penetration tests (often referred to as “pen-tests”) can play a crucial role as they simulate malicious hackers’ intrusions, thus locating the system's weak points. Pen-tests are authorized attempts to break into a system in the same way malicious hackers would do [26]. To gain this knowledge, someone who thinks like a malicious hacker is needed. Even better, hackers themselves should conduct pen-tests, ethical hackers. Pen-tests can help healthcare facilities to be aware of weaknesses and to fix them before a malicious hacker finds them.

**Fig. 1:**
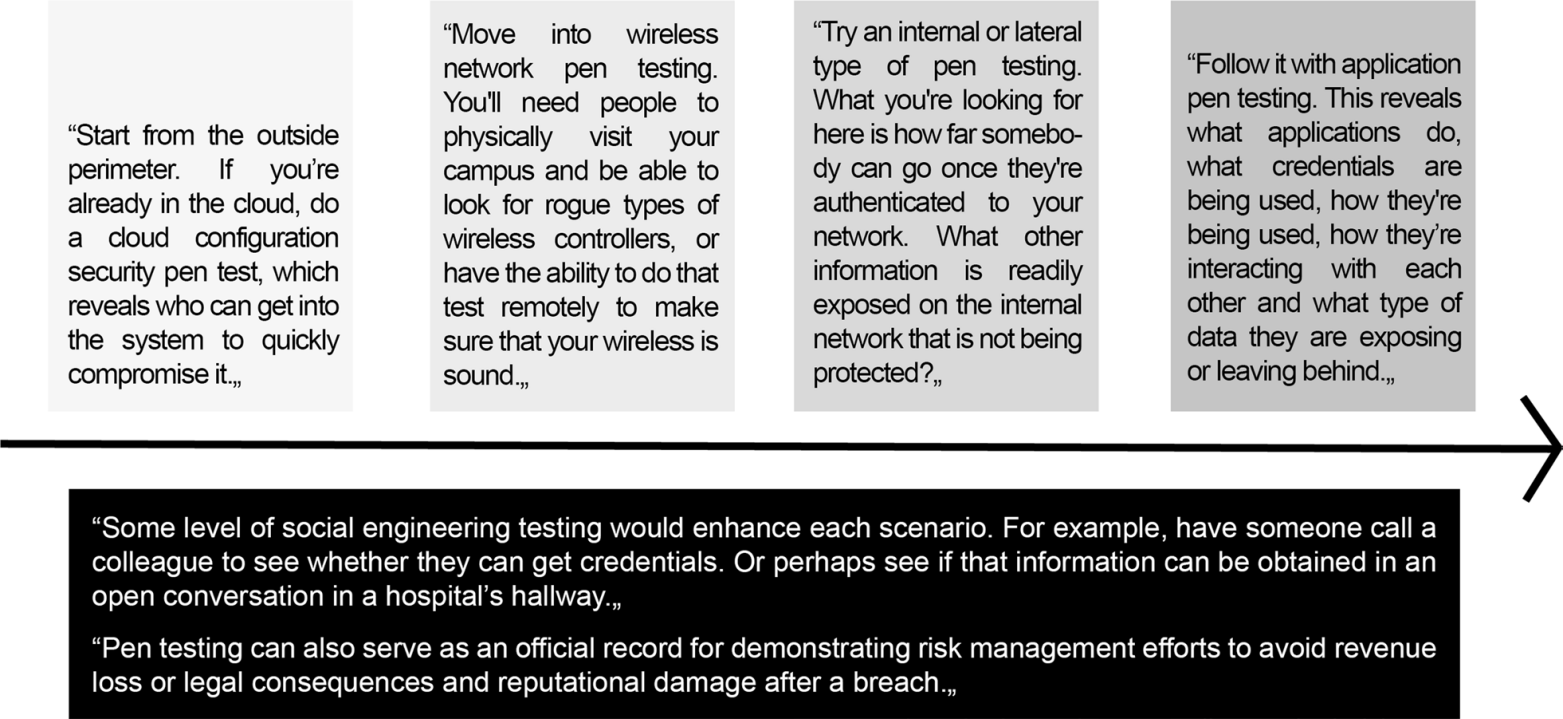
Example of a four-step approach pen-test for healthcare. Figure based on: Gregory, M. Tabletop Exercises and Penetration Testing: What Health Systems Need to Know, in HealthTech Magazine, 2021. https://healthtechmagazine.net/article/2021/12/tabletop-exercises-and-penetration-testing-what-health-systems-need-know. Accessed on 30 Sep 2022

It is fundamental to understand that the pen-test is a common service provided by reputable companies. Pen-testers are therefore employed or have a contractual relationship with the company that has been hired. It is true that pen-testers often resort to the same techniques and tools as malicious hackers, which can sometimes be controversial (e.g. social engineering strategies, such as phishing and USB drops^2^) [27]. But the goal is always to conduct realistic simulations to efficiently bolster cybersecurity [26]. Although technically they would still break into systems, pen-testers would be under the obligations of a contract that explicitly establishes boundaries and prohibited practices. They must have written permission before conducting pen-tests, and during the assessment, they must stay within the scope of the employers’ expectations, which must have been previously disclosed and discussed. It is also essential that they transparently communicate all the findings and provide a detailed transcript of their actions [28]. Contemporarily, there is explicit consent on the part of the employer. The establishment of ad hoc legal contracts is a necessary step to support the employment of pen-testers in healthcare cybersecurity (Fig. 1).

Ethical hacker certification exists and is becoming increasingly important [12]. At the same time, the possibilities to get certified are also increasing: there are already many institutions that offer certification, both theory and experience-based. Additionally, many universities are offering ethical hacking courses [12]. These certificates can be very convenient in the hiring process for pen-testers.

Hiring companies that provide pen-test services is not the same thing as allowing anonymous ethical hackers to try and hack into a healthcare facility. While rewarding and bug bounty programs can work perfectly fine for other sectors, in healthcare this may cause controversies. As previously seen, the healthcare sector is particularly vulnerable and its data is very sensitive. Collaborating with companies seems a better path as this allows to negotiate the details of the service provided and establish specific contracts; this also entails the employer’s explicit consent to the pen-test. At the same time, the identity of pen-testers is known and the company can be held liable if damages occur.

There is therefore a difference between pen-testers and ethical hackers that is now necessary to elucidate. While these two terms are often used interchangeably, “ethical hacker” is rather an umbrella term that includes all hacking methodologies and techniques [29, 30]. It is correct to say that pen-testers are ethical hackers, however not all ethical hackers are pen-testers. Pen-testing is a very planned process that requires all necessary permissions; although it mimics real-life cyberattack scenarios, it is a helpful and non-harmful process [30]. Pen-testers are certified professionals hired by companies under legal contracts, instead, this is not a requirement for ethical hackers that usually act on a voluntary basis and through bug bounty programs; these do not involve contractual relationships and informed consent procedures. The range of attack vectors and attack types for pen-testers is limited, whereas this limitation is not present when referring to ethical hacking. While usually adhering to high standards of behaviour [31], the lack of limitations on the available hacking techniques may sometimes cause collateral damage (e.g. computer downtime or data breaches).

Acknowledging the subtle difference between ethical hackers and pen-testers is important for employing them in healthcare cybersecurity. Since pen-testers still fall under the “hackers” category, understanding what hacker ethics is and who ethical hackers are can be relevant when deciding to hire their services.

## Understanding hackers and their ethics

Hacking began in the 1970s and is defined as the “unauthorized intrusion into a computer system” [32]. This definition includes both the practice of malicious hacking and ethical hacking. The difference lies in the hacker’s intent: if the purpose of hacking is just for the challenge, the thrill, and finding (and reporting) leaks in the security, but without stealing money or disseminating data, then that hacker is called a “white hat”, or an ethical hacker. Note that hacking for the thrill or challenge alone would not constitute ethical hacking in the absence of reporting. There is a grey area where someone is neither an ethical hacker nor a malicious one; they are what can be called a “grey hat”: morally ambiguous hackers that do not fully adhere to ethical hackers’ principles but whose actions are not fundamentally guided by malicious intentions [28, 33]. Instead, if the aim is the hacker’s financial gain and disruption, we are faced with a “black hat”, namely a malicious hacker. Nonetheless, it is not always so simple to differentiate and the same hacker can sometimes act in both ways or later “convert” to ethical hacking. Some argue that it is wrong to fit hackers into a moral binary, in which they are either heroes or villains [34].

Besides the difficulty of categorizing every hacker with certainty, there is the issue that even hacking “for the good” can be punishable. This causes many ethical hackers to avoid reporting vulnerabilities for fear of legal repercussions^3^ [35]. It also contributes to hackers’ willingness to work in the shadows and consequently creates a distorted perception of hacking practices. Media coverage that portrays them in dark hoodies in dark rooms at night further contributes to this misconception. The term “hacker” itself presents negative and pejorative connotations, stigmatizing a widely varied group [28, 36]. In an effort to collaborate with ethical hackers (and to professionalize them as pen-testers, particularly in the healthcare sector), a first fundamental step is to acknowledge the prejudices and narratives surrounding their practice. A second step would entail recognizing the existence of different categories of hackers. Lastly, better understanding hacker ethics could address some controversies and concerns.

Considering hacker ethics is useful for better understanding ethical hackers and their values. Sooner or later, hackers are confronted with ethics. Even if hacking is not primarily an ethical issue, most hackers come to a point where they have to face some ethical questions, hence, there is a certain connection between hacking and ethics [37]. Hacker ethics is a type of personal ethics, therefore every hacker has a unique understanding of its values. In fact, some claim that “there is no hacker ethics. Everyone has his own” [34]. Despite the lack of unitary hacker ethics, the many hacker codes present numerous similarities [38, 39]. Indeed, they all somehow share liberalistic ideals. For example, they endorse open-source projects and are very privacy-aware. What differs is how they interpret and defend these ideals. This difference can be well-illustrated by the positive and the negative understanding of “freedom”.

In its positive connotation, freedom invokes free and open access to information with the pedagogical goal of equally allowing humans to educate themselves [34]. What matters is to advance human knowledge, make sure that it is available to everyone, and encourage cooperation [37]. From this perspective, mechanisms to privatize and monetize information and software constitute a barrier and are considered unethical [34]. Copyright laws corrupt freedom since information is not ownable property. Sharing information would then be a moral imperative. However, this does not call for the elimination of all barriers: it is important to maintain and enforce privacy measures. This is a freedom that values learning, community, sharing, and equal opportunities. It aims to advance human knowledge and bridge the current information gap. For this reason, the focus is on the liberalization of knowledge and open-source software, rather than on the notion of privacy, although deemed extremely important.

The negative sense of freedom stands close to anarchistic ideals and can be intended as “freedom from everything”. It greatly values privacy and often leads to acts of civil disobedience to protect it [34]. It is antagonistic to institutionalization and surveillance measures. The focus is on self-determination and non-interference of others. Its primary values are individual autonomy, self-reliance, and, of course, individual privacy. While positive freedom emphasizes community welfare, negative freedom is focused on individuality.

Hacker ethics is neither dichotomic nor unitary; it entails a different, and sometimes contradictory, understanding of values. However, as has been previously observed, there are commonalities and similarities. It is noteworthy that it revolves around two values: freedom and privacy. Although distinctively interpreted, they constitute the core of hacker ethics. Hackers’ actions often emanate from different interpretations of these two values. However, adherence to hacker ethics does not imply that their actions would be deemed morally good by society: some hackers may advocate their ethics by stealing confidential information and disseminating it.^4^ Although black hat hackers’ actions are generally unquestionably unethical, with grey hat hackers the morality of some actions can be debatable (for example, grey hat hackers that report vulnerabilities often threaten the owner of the hacked system to publicly reveal it, hence enormously exposing the system to malicious hackers’ attacks, in case it will not be timely patched [28]). Therefore, for more safely employing pen-testers in healthcare cybersecurity, it is necessary to re-think hacker ethics, and in particular the understanding that ethical hackers have of it, as something else than just a personal ethics that is subject to an immense variety of strands and interpretations.

Pen-testers comply with a specific interpretation of hacker ethics, namely the one that includes and prioritizes respect for individuals’ privacy. This entails that pen-testers do not disseminate or leak data. They also do not intend to cause damage when hacking into systems, nor do they download, modify, or disseminate the data. Their intent is rather to find vulnerabilities and appropriately report them. Therefore, they work towards the establishment of a safer cyber environment. They are institutionalized (through regular employment contracts and certifications) and confine their activity within the law [28]. It is true that pen-tests often resort to the same techniques and tools as malicious hackers, but the goal is always to conduct realistic simulations to efficiently bolster cybersecurity without disrupting the workflow [26]. Following the present description of pen-testing practice, it seems possible to consider their ethical hackers’ ethics as a sort of professional ethics, beyond that of personal ethics. This would allow for a two-fold benefit: it would be possible (and recommended) to draft an international code of ethics that can less arbitrarily define and describe moral principles, standards, expectations, and best practices; also as a consequence, it would facilitate the regulation of their practice and allow punitive measures when said code is disrespected. When professionals disregard their code of ethics they lose the right to practice. Equally, when pen-testers are intentionally violating privacy norms by, for example, breaching data or damaging infrastructures, they could be excluded from the cybersecurity field. This can be enforced with pen-testers as they are regularly employed, and controlling and sanctioning their behaviour can be simpler than with ethical hackers in general. However, as of now, there is no official ethics of conduct for pen-testers. At a professional level, the absence of an ethical code is surprising. A similar code would be a great advantage for further promoting the service of pen-testing, particularly in sensitive sectors such as healthcare. Following the present conceptualization of hacker ethics, it seems possible to consider the employment of ethical hackers as pen-testers in healthcare cybersecurity, with the recommendation of establishing an official code of ethics for their practice.

## Conclusion

Cyber threats to healthcare are an unavoidable new reality. However, there are ways to strengthen healthcare cybersecurity. For this sector, cybersecurity is not only about protecting data: health data is particularly sensitive and protecting it equals maintaining patients’ safety, privacy, and trust [7]. While pen-tests alone will not, and cannot, solve the cybersecurity vulnerabilities of healthcare, they surely can constitute a further measure to bolster it. In this paper, it has been shown how pen-tests are compatible with the healthcare sector and can be advantageous. Other cyber-hygiene steps are needed, among them: removing legacy software like Windows XP and promoting best practices.

Pen-tests can greatly contribute to cybersecurity. It seems that the best way to employ ethical hackers as pen-testers in healthcare is to hire a company providing pen-test services. Hacker ethics, in general, is not particularly relevant for identifying ethical hackers; rather, a particular understanding of this ethics, emphasising privacy and data protection, could help set professional standards for pen-testers. Therefore, it is recommended to work on an official, national or international, code of ethics for this profession. In addition, considering hacker ethics can raise awareness of the prejudices about the hacking practice and address narratives of fear. For this reason, is it important to acknowledge the variety of hackers and their ethics.

Accepting ethical hackers, especially pen-testers, into our society can bring significant benefits. Firstly, they can greatly contribute to cybersecurity in general, and particularly in the healthcare sector with pen-testers. However, relying solely on pen-tests will not solve the cybersecurity issues of healthcare: other cyber-hygiene measures still need to be implemented and improved. Secondly, they could help address the labour shortage affecting the cybersecurity industry. Again, this is valid not only for healthcare cybersecurity; however, the limited financial resources of this sector constitute a limitation in the employment of pen-testers. Eventually, the prime goal of healthcare is to protect human life and health; balancing financial resources to reinforce the cybersecurity of this sector can contribute to that goal.

## Data Availability

Not applicable.

## Abbreviations

AI: Artificial intelligence
EHR: Electronic health records
Pen-tests: Penetration tests
Pen-testers: Penetration testers
Pen-testing: Penetration testing
UVM: University of Vermont

## Footnotes

1 AI could also be used to enforce healthcare cybersecurity by detecting suspicious activity within the facility network, hence addressing the difficulty human experts face in detecting intrusions. Indeed, it often takes weeks, if not months, for humans to detect breaches and contain the damage. Cybersecurity staff are often overwhelmed by threat alerts and report risks. AI could free humans from this burden by automatically checking all the alerts; although humans would still need to evaluate AI’s warnings, these tools can render their jobs more efficient. However, monitoring the facility network might have privacy implications if employees are surveilled at their workplace. Therefore, cybersecurity AI should be carefully implemented to avoid undesirable consequences, such as privacy invasions.

2 Phising and USB drops are only two examples of the many social engigneering methods that pen-testers can use. Phising refers to emails sent with the intent to deceive the receiver; they usually include malicous links or requests for sensitive and private information. USB drops consist in leaving a USB stick in a common area (e.g. the hospital secretariat); this USBs typically contain malicous softwares that, when plugged into the hospital IT infrastructure, allows access to that system.

3 Some companies (e.g. Google, Microsoft, Facebook) and governments (e.g. in the Netherlands) already adopted approaches and legislation that allow ethical hackers to report vulnerabilities, while protecting them form legal repercussion; this protection is crucial to continuing collaboration with ethical hackers to bolster cybersecurity. Lacking this legal protection, many vulnerabilities could go unreported out of fear of legal repurcussions, hence leaving open a possibility for malicous hackers to exploit said vulnerability. The discussion pertaining to the boundaries and modalities of the practice is still ongoing; it appears that as long as no harm is done to the system and the data, nothing is leaked, and the vulnerability is appropriately reported, the ethical hacker should not be prosecuted. It nonetheless remains difficult to assess what “appropriately reported” means and whether a copy of the data was made. In the meanwhile, new regulations are being issued (e.g. in May 2022 the US Department of Justice ruled that ethical hackers will no longer be prosecuted under the Computer Fraud and Abuse Act) that aim to tackle this delicate situation.

4 This is often the case of hacktivists, that pursue ideological objectives (be they political, religious, or pertaining personal values and motivations) careless of their means’ morality, repercussions and adequatness.
